# Environmental Concerns, Environmental Policy and Green Investment

**DOI:** 10.3390/ijerph14121570

**Published:** 2017-12-13

**Authors:** Xuexian Gao, Haidong Zheng

**Affiliations:** School of Economics & Management, China University of Petroleum (East China), Qingdao 266580, China; gaoxuexian1569@upc.edu.cn

**Keywords:** green technology, green investment, environmental concern, environmental tax, low-emission

## Abstract

Environmental regulators often use environmental policy to induce green investment by firms. However, if an environmental policy fails to exert a long-run effect on regulating the economic agents’ behavior, it may be more reasonable to think of the firm as the leader in the game, since the investment in green technology is usually a strategic decision. In this paper, we consider a three-stage Stackelberg game to address the interaction between a profit-maximizing firm (Stackelberg leader) facing emission-dependent demand, and the environmental regulator (Stackelberg follower). The firm decides on the green technology level in the first stage of the game based on its understanding of the regulator’s profits function, especially an environmental concern that is introduced as an exogenous variable. In the current research, we show that high levels of the regulator’s environmental concerns do not necessarily lead to the choice of green technology by the firm, and green investment level depends on the combined effects of the market and operational factors for a given level of the regulator’s environmental concerns. The result also shows that increasing environmental awareness amongst the consumers is an effective way to drive the firm’s green investment.

## 1. Introduction

The issues that are related to the environment have received a large amount of attention over the last several years. In China, for example, once the government’s main concern was the economic growth, which in turn caused serious environment damage. Nowadays, policy makers have been aware of the environmental issues and are introducing different environmental protection policies to regulate the behavior of economic agents in green technology investment. Some Chinese scholars [1] have argued that the regulator should adopt a dynamic tax/subsidy system. However, this results in the failure of the policy to extend its commitment power to the long run and might cause the economic agents to behave opportunistically [2,3]. As an instance, before 2018, the Chinese government regulated the business green behavior by levying pollutant discharge fee, which is without a national uniform standard and is completely determined by local regulators being in charge of the office only temporarily. As a major financial resource, local regulators in economically underdeveloped areas, especially in Midwest, determine the annual budget expenditure of environmental protection, according to the pollutant discharge fee. On 25 December 2016, an environmental protection tax law was adopted by Chinese government and will be implemented since 1 January 2018. However, the tax law gives only a reference range for unit emission tax, and there is still not a uniform standard. Thus, if the environmental policy fails to exert a long-run commitment power on regulating the economic agents’ behavior, it may be more reasonable to think of the firm as the leader in the game, since the investment in green technology is usually a strategic decision. Dewit and Leahy [3] proposed a game model, in which the firm is the leader to make its decision to affect the regulator’s policy. They focused on investigating the propensity of two tax systems (i.e., uniform and differentiated tax systems) to create distortionary opportunistic behavior.

The goal of this paper is to address the interaction between a profit-maximizing firm, which emits undesirable pollutant as a natural by-product of its production process, and the environmental regulator. To do so, we consider a three-stage Stackelberg game, in which the firm can observe the environmental concern of the regulator, and must choose its emissions-reducing technology in the first stage of the game. Then, the regulator decides on the tax/subsidy level in response to the green technology investment of the firm with a trade-off between economic and environmental profit. In the final stage, the firm chooses its price (or output) of its product. The market demand is assumed to be decreasing with price and emission. To measure the degree of environmental concern, we introduce the parameter of “decision weight” in the regulator’s profit function.

The remainder of the paper is organized as follows. The existing literature is reviewed in Section 2. The model is formulated in Section 3. Section 4 studies the firm’s and the regulator’s problem in a three-stage Stackelberg game, where some numerical examples are also provided. Finally, Section 5 summarizes the paper and discusses future research possibilities and requirements. An appendix detailing the model derivation is also accompanied.

## 2. Literature Review

This paper is related to two streams of literature: operation management and economics.

As with the operation management, an important research domain regarding environmental problems is remanufacturing [4,5,6,7,8,9,10]. Raz et al. [9] discussed the problems of environmental innovations in product and process design. They focused on where such design changes could be economically most effective to the firm and what the environmental consequences of these changes would be. They considered a profit maximizing firm who needed to make a decision on the production quantity, as well as its environmentally focused design efforts. Their results showed that the total environmental impact could either increase or decrease due to the increased production quantities. They identified the conditions for such cases by looking at the environmentally focused design efforts that are needed to compensate for the increase in production. Esenduran and Kemahlıoğlu [6] discussed the problem of compliance with product take-back regulation, under which the firms finance the collection and treatment of their end-of-life products. They compared two compliance schemes, collective compliance scheme, and individual compliance scheme, with respect to the costs they impose on firms and environmental benefits. They identified conditions under which collection rates are higher when firms comply individually and recyclability levels are higher when firms comply collectively and allocate costs with respect to market shares.

Much of the literature on operation management has considered social costs that are caused by the carbon emissions into the supply chain optimisation model [11,12,13,14]. Tseng and Hung [14] propose a strategic decision-making model considering both the operational costs and social costs to evaluate carbon dioxide emissions and operational costs under different scenarios in an apparel manufacturing supply chain network. Their results showed that the amount of the emission decreased with the social cost rate of carbon dioxide emissions. Sarkis [13] introduced a supply chain optimization modal to examine the possible economic and environmental trade-offs for various carbon-pricing and fuel-pricing scenarios. Rezaee et al. [12] presented a two-stage stochastic programming model to solve a discrete location problem and to determine the optimal material flows and the number of carbon credits/allowances traded. According to their results, the supply chain configuration can be highly sensitive to the probability distribution of the carbon credit price.

On the other hand, in the economics literature, many publications have focused on the relationship between policy intervention (such as government subsidies or environmental tax) and green technology investment (or other green activities). These research studies could be reviewed as follows. Wang et al. [15] modeled two scenarios as decentralized remanufacturing supply chains, when considering the low environment consciousness of customers in developing countries, like China. They assumed that the manufacturer was the Stackelberg leader and the government offered subsidy to the remanufacturer to incentivize green activities. Gsottbauer and Jeroen [16] developed a theoretical model to analyze optimal environmental policy when pollutive consumption is sensitive to consumption by others and commercial advertising. Lee et al. [17] analyzed the investment status of green technology R&D in Korea, through which the policy direction for improving investment efficiency and efficient distribution of green technology R&D was proposed. Lambertini and Tampieri [18] proposed a model of environmental overcompliance, where firms set the environmental quality of their products and compete in quantities, while the government imposes an environmental standard with the aim of maximizing welfare. They showed that all firms overcomply if the environmental impact of production is sufficiently low; otherwise, unilateral overcompliance emerges by the firm with higher environmental quality. Espínola and Muñoz [19] investigated the conditions under which the firm profits are enhanced by environmental policy, and showed that in contrast to the common belief, inefficient firms may support environmental regulation when their production is significantly polluting. Bian et al. [20] explored the effects of environmental taxationt on distribution channel selection decisions.

Based on the studies that are discussed above, this paper is closely related to several papers as follows. Krass et al. [21] studied several important aspects of using environmental taxes or pollution fines to motivate the choice of innovative and “green” emissions-reducing technologies. They assumed that the firm was purchasing technology in the market and was facing discrete technology choices. Different from Krass et al. [21] and Raz et al. [9], Arguedas et al. [22] and Bi et al. [23] assumed that a firm’s cost of reducing emissions was a continuous twice-differentiable function, which implied that a continuum of technologies was available. However, the current study combine this two stream by assuming that the firm incurs both one-time fixed cost and variable operating cost, whereas the cost function is continuous.

As for the Stackelberg game, most of the researches have assumed the environmental regulator as the leader to set the tax level and profit-maximizing monopolistic firms as follower to response to it [16,21,23]. However, Isik [2] showed that uncertainty about government policies significantly impacts the green investment decision of economic agents. Dewit and Leahy [3] argued that many environmental policies, including emission taxes in the real world, typically have commitment power in the short run, but fail to extend that power to the longer run. Based on these arguments and examples in Chinese context mentioned above, we assume the firm as the leader to set his strategic decision of green technology investment level first, and the regulator as the follower to set the tax (or subsidy) level in response to it.

The regulator’s profit function is an important aspect to characterize a specific decision scenario. For instance, some researchers have assumed that the regulator is not considering tax revenue as part of social welfare [24,25], while many others have believed the opposite [21,26]. Meanwhile, following the traditional definition in economics, many researchers have assumed that the social regulator maximizes the social welfare, which consists of firm profits, consumer surplus, and other externalities [21,24,26]. However, many other studies have modeled the regulator’s profit function based on specific issues the study concerned. For example, Dewit and Leahy [3] assumed the regulator’s profit consisited of the firm’s profits, the environmental damages, and emission taxes in the study of how the tax system would create distortionary opportunistic behaviour. Arguedas et al. [22] presented a Stackelberg differential game to study the dynamic interaction between a polluting firm and a regulator who sets pollution limits overtime. In their work, the firm’s profits, the environmental damages, and the social cost of enforcing the pollution limit were used as a measure of social welfare. This paper, for simplicity, follows Dewit and Leahy [3] to model the regulator’s profit function, which does not include consumer surplus and focuses mainly on the concerned issues. Nevertheless, but the current study is different from the above-mentioned works in that we considered the “decision weight” in the regulator’s profit function based on the trade-off concern between economic and environment in an emerging country like China.

In summary, the present paper would be differentiated from the aforementioned literature in many dimensions as follows: we assumed the firm as the Stackelberg game leader to make decision on green technology investment first, which, in turn, would influence the regulator’s tax/subsidy rate decision making. Besides, the focus of this study was on investigating the effect of the regulator’s decision weight of environmental protection, as well as the market environmental consciousness, on the firm’s green technology investment. The framework we used herein, is closer to the works in operations that can help to capture the nature of firm’s technology investment decisions.

## 3. The Model

We considered a three-stage Stackelberg game between a regulator and a firm. Based on the argument of Dewit and Leahy [3], the firm’s green technology investment is typically a long-run strategic decision and entails a great deal of commitment values because of its irreversibility. While the regulator has commitment power in the short run to set the environmental tax (or subsidy) rate, it cannot extend that commitment power to the longer run. Thus, we assumed that the firms make its long-run investment decisions in the first stage, the regulator sets its emission tax or green subsidy rate in the second stage, and the firm chooses retail price, which has very little commitment values in the final stage.

We assumed the firm face a market with linear demand function D=A−αp, where A>0 is the market scale (i.e., the potential demand or market size), α>0 is the elasticity of demand, and D is the realized demand under retail price p. Moreover, the market is assumed to be low-emission sensitive. As already mentioned in many studies, consumers are willing to pay extra for low-pollutant products [27,28,29]. So the firm may have an incentive to adopt green technology in the production process to lower the amount of pollutants emitted. Let l be the technology level that refers to, for example, a firm’s investment decision to reduce pollutants emitted during manufacturing process. For a fixed l, the emissions per unit of product will decrease by γl, where γ>0, is the pollution-reducing effect of l. Let $e_{0}$ be emission per unit of product without low-emission effort and β be the low-emission preference of consumers. Then, the final demand function would be given by

(1) $$D\left(p,l\right)=A-\alpha p-\beta \left(e_{0}-\gamma l\right),$$

where we made the assumption that $e_{0}-\gamma l\geq 0$ (i.e., $l\in \left[0,\frac{e_{0}}{\gamma }\right]$), meaning $l=\frac{e_{0}}{\gamma }$ is the highest level of green technology to eliminate the pollutant emitted.

For the sake of simplicity, the firm’s manufacturing cost per unit of product without green technology was not taken into account to focalize the problem in the pollutants emission factor. Similar to Raz et al. [9], Arguedas et al. [22] and Bi et al. [23], we assumed that the one-time fixed (purchase, acquisition, installation, and other relevant factors) green technology investment cost was a quadratic function as $C\left(l\right)=c\frac{l^{2}}{2}$, where index 2, which was taken as a fix number for simplicity, is a measure of the complexity of the green technology to develop [30]. We assumed that the production cost per unit increased by θl because of adopting the green technology and θ is unit cost increase coefficient.

Then, the firm’s profit function could be given by

(2) $$\Pi_{f}=\left(p-\theta l-\left(e_{0}-\gamma l\right)t\right)D\left(p,l\right)-C\left(l\right),$$

where t is the environmental tax(or subsidy) per unit of pollutants emitted into the environment. We assumed the regulator subsidizes the firm when t<0; otherwise, environmental tax is charged. In other words, in our research, the environmental policy was regarded as continuous choices from subsidy to tax.

The environmental regulator can be assumed as a system-wide decision-maker who needs to make a trade-off between economic development and its environmental damage (i.e., the regulator needs to be simultaneously concerned about the firm’s profits and the environmental damages). So, the regulator’s profit would be given by

(3) $$\Pi_{r}=\left(1-\lambda \right)\Pi_{f}+\lambda \left(\left(e_{0}-\gamma l\right)t-\varepsilon {\left(e_{0}-\gamma l\right)}^{2}\right)D\left(p,l\right),$$

where λ(0<λ<1) denotes the decision weight(or environmental concern) the regulator adopts in the environment protection decision. A higher λ implies a higher degree of environmental concern/awareness of the regulator. Meanwhile, the first component of $\Pi_{r}$ is the firm’s profits multiplied by (1−λ) standing for economic concern of the regulator, and the second component, $\lambda \left(\left(e_{0}-\gamma l\right)t-\varepsilon {\left(e_{0}-\gamma l\right)}^{2}\right)D\left(p,l\right)$, denotes the environmental welfare. Similar to Arguedas [22], we define $\varepsilon {\left(e_{0}-\gamma l\right)}^{2}$ as the environmental damage that us caused by unit of pollutants emitted, where ε specifies the degree of environmental hazard of the firm’s physical production process and the associated pollutant.

As mentioned above, the firm and the regulator play a three-stage game, where the firm’s green technology investment is a long-run strategic decision because of its irreversibility and can be used to influence the tax/subsidy rate imposed on the firm by the regulator [3]. Therefore, in the first stage of the game, the firm makes its long-run investment decision (i.e., the technology level), denoted by l. The regulator sets the environmental tax (or subsidy) rate t at the second stage and the firm chooses retail price p in the final stage.

## 4. Analysis of the Game

Using backward induction, we first turned to the final stage (Section 4.1) where the firm determines the retail price. Subsequently, the regulator sets the optimal emission tax (or subsidy) (Section 4.2). Finally, we derived the firm’s investment levels under the tax rate (Section 4.3). We will show that how the regulator’s environmental concerns influence the green investment decision of the firm, and, in turn, the environmental policy in different scenarios.

### 4.1. The Optimal Market Response 

In stage three, the firm maximizes profit with respect to the selling price p for a given t. Then, the firm’s problem in this stage can be formulated as

(4) $$\mathrm{Maximize}\;\Pi_{f}\left(p\left(t\right)\right)=\left(p-\theta l-\left(e_{0}-\gamma l\right)t\right)D\left(p,l\right)-C\left(l\right)$$

Using first-order conditions, the optimal price is given by

(5) $$p^{*}\left(t\right)=\frac{A+\alpha \theta l+\left(\alpha t-\beta \right)\left(e_{0}-\gamma l\right)}{2\alpha }$$

### 4.2. The Optimal Tax Rate

**Proposition** **1.** *The regulator cannot find an optimal environmental tax rate* $t^{*}$ *when* $0\leq \lambda <\frac{1}{3}$ *.*

See Appendix A for the proof.

Based on Proposition 1, the case of $0\leq \lambda <\frac{1}{3}$ was ignored in this research.

In another case when $\frac{1}{3}\leq \lambda \leq 1$, $\Pi_{r}$ is concave with a unique maximum at:

(6) $$t^{*}\left(l\right)=\frac{\left(2\lambda -1\right)\left(A-\alpha \theta l-\beta S_{1}\right)+\lambda \alpha \varepsilon S_{1}^{2}}{\left(3\lambda -1\right)\alpha S_{1}}\;$$

By substituting (6) into (5), we have:

(7) $$p^{*}\left(l\right)=\frac{\left(5\lambda -2\right)\left(A-\beta S_{1}\right)+\lambda \alpha \left(\theta l+\varepsilon S_{1}^{2}\right)}{2\alpha \left(3\lambda -1\right)}.\;$$

Obviously, the firm will choose to produce only if the profit is positive, which implies that both the realized demand D(p(t(l)),l), and the price markup must be positive.

**Theorem** **1.** *To enable production, there must exist at least a* l *to satisfy the necessary and sufficient conditions as follows:*

(8) $$\{\begin{array}{c} D\left(p^{*}\left(l\right),l\right)=A-\alpha p^{*}\left(l\right)-\beta \left(e_{0}-\gamma l\right)>0\\ p^{*}\left(l\right)-\theta l-\left(e_{0}-\gamma l\right)t^{*}\left(l\right)>0 \end{array}$$

*Otherwise, the firm will gain no profit from production and will choose not to produce.*

For notational convenience, we defined $S_{2}\equiv A-e_{0}\beta -\alpha \varepsilon e_{0}^{2}$ and $S_{3}\equiv \beta \gamma -\alpha \theta +2\alpha \varepsilon e_{0}\gamma$. We also assumed $S_{2}>0$，$S_{2}\gg S_{3}$ and $A\gamma -\alpha \theta e_{0}>0$ considering A is a big enough number. Thus, if (8) is satisfied, then $-\alpha \varepsilon \gamma^{2}l^{2}+S_{3}l+S_{2}>0$, which needs the following condition hold.

(9) $$0\leq l\leq \frac{S_{3}+\sqrt{S_{3}^{2}+4\alpha \varepsilon \gamma^{2}S_{2}}}{2\alpha \varepsilon \gamma^{2}}\;.$$

As mentioned in Section 3, l must also satisfy $0\leq l\leq \frac{e_{0}}{\gamma }$. Therefore, we first needed to confirm the domain of l by comparing $\frac{e_{0}}{\gamma }$ with $\frac{S_{3}+\sqrt{S_{3}^{2}+4\alpha \varepsilon \gamma^{2}S_{2}}}{2\alpha \varepsilon \gamma^{2}}$.

We had

(10) $$\frac{S_{3}+\sqrt{S_{3}^{2}+4\alpha \varepsilon \gamma^{2}S_{2}}}{2\alpha \varepsilon \gamma^{2}}-\frac{e_{0}}{\gamma }=\frac{\beta \gamma -\alpha \theta +\sqrt{{\left(\beta \gamma -\alpha \theta \right)}^{2}+4\alpha \varepsilon \gamma \left(A\gamma -\alpha \theta e_{0}\right)}}{2\alpha \varepsilon \gamma^{2}}>0\;,\;$$

which implies that (9) is ensured when $l\in \left[0,\frac{e_{0}}{\gamma }\right]$.

Based on Theorem 1, we had the following propositions.

**Proposition** **2.** *The regulator’s optimal environmental tax rate* $t^{*}\left(l\right)$ (*when* $t^{*}\left(l\right)>0$) *is increasing in its environmental concern* λ. *The regulator’s optimal environmental subsidy rate* $\left|t^{*}\left(l\right)\right|$ (*when* $t^{*}\left(l\right)\leq 0$) *is decreasing in its environmental concern* λ*.*

The proof for Proposition 2 is obvious. Differentiating $t^{*}\left(l\right)$ with respect to λ yields $\frac{\partial t^{*}}{\partial \lambda }=\frac{-\alpha \varepsilon \gamma^{2}l^{2}+S_{3}l+S_{2}}{{\left(3\lambda -1\right)}^{2}\alpha S_{1}}>0$.

**Proposition** **3.** *The firm’s optimal selling price* $p^{*}\left(l\right)$ *is increasing in the regulator’s environmental concern* λ *by satisfying Theorem 1 .*

The proof for Proposition 3 is also straightforward.

### 4.3. The Optimal Green Technology Investment

In this section, we explore the impacts of the regulator’s environmental concern λ on the firm’s technology level l decision-making considering different scenarios. We defined $\bar{l}\equiv \frac{e_{0}}{\gamma }$ as the upper bound of l. Substituting (6) and (7) into (2), the maximization problem for the firm could be formulated as:

(11) $$max\Pi_{f}\left(l\right)=\frac{{\left(A-\alpha \theta l-\beta S_{1}-\alpha \varepsilon S_{1}^{2}\right)}^{2}\lambda^{2}}{4\alpha {\left(3\lambda -1\right)}^{2}}-c\frac{l^{2}}{2}\;s.t.:\;l\in \left[0,\bar{l}\;\right]$$

As in the following section, we investigated decision-making in different cases.

#### 4.3.1. Sub-Case A: It is Favorable to Adopt Green Investment (βγ−αθ≥0)

(1) Sub-case A.1 $\sqrt{\frac{\left(\beta \gamma -\alpha \theta \right)\left(A\gamma -\alpha \theta e_{0}\right)}{2\alpha ce_{0}}}<2$

Defining $E_{1}\equiv \left\{\lambda |\frac{1}{3-\sqrt{\frac{\left(\beta \gamma -\alpha \theta \right)\left(A\gamma -\alpha \theta e_{0}\right)}{2\alpha ce_{0}}}}<\lambda <1\;\right\}$, then we had the following proposition.

**Proposition** **4.** *When*  βγ−αθ≥0 *(i.e.,* $\frac{\beta }{\alpha }-\frac{\theta }{\gamma }\geq 0$ *) and* $\sqrt{\frac{\left(\beta \gamma -\alpha \theta \right)\left(A\gamma -\alpha \theta e_{0}\right)}{2\alpha ce_{0}}}<2$ *,* *there exist a* $l^{*}$ *such that* $l^{*}\in \left[0,\bar{l}\right)$ *to maximize the firm’s profit by satisfying Theorem 1 and* $\lambda \in E_{1}$ *.*

See Appendix B for the proof.

(2) Sub-case A.2 $\sqrt{\frac{\left(\beta \gamma -\alpha \theta \right)\left(A\gamma -\alpha \theta e_{0}\right)}{2\alpha ce_{0}}}\geq 2$

In this case, we had the following proposition.

**Proposition** **5.** *When*  βγ−αθ≥0 *(i.e.,* $\frac{\beta }{\alpha }-\frac{\theta }{\gamma }\geq 0$ *) and* $\sqrt{\frac{\left(\beta \gamma -\alpha \theta \right)\left(A\gamma -\alpha \theta e_{0}\right)}{2\alpha ce_{0}}}\geq 2$ *, the firm will always choose the highest level of green investment (i.e.,* $l^{*}=\frac{e_{0}}{\gamma }$ *)* *by satisfying Theorem 1 and* $\lambda \in \left[\frac{1}{3},1\right]$ *.*

See Appendix C for the proof.

#### 4.3.2. Sub-Case B: It Is Not Favorable to Adopt Green Investment (βγ−αθ<0)

(1) Sub-case B.1 $\frac{\theta }{\gamma }-\frac{\beta }{\alpha }<2\varepsilon e_{0}$

In this case, we had the following proposition.

**Proposition** **6.** *When* βγ−αθ<0 *and* $\frac{\theta }{\gamma }-\frac{\beta }{\alpha }\leq 2\varepsilon e_{0}$ *, t* *here exist a* $l^{*}$ *such that* $l^{*}\in \left[0,\bar{l}\right]$ *to maximize the firm’s profit by satisfying Theorem 1 and* $\lambda \in \left[\frac{1}{3},1\right]$ *.*

See Appendix D for the proof.

(2) Sub-case B.2 $\frac{\theta }{\gamma }-\frac{\beta }{\alpha }\geq 2\varepsilon e_{0}$

**Proposition** **7.** *When*  βγ−αθ<0 *and* $\frac{\theta }{\gamma }-\frac{\beta }{\alpha }\geq 2\varepsilon e_{0}$ *, the firm will never invest in any green technology given any* $\lambda \in \left[\frac{1}{3},1\right]$ *.*

See Appendix E for the proof.

**Figure 1:**
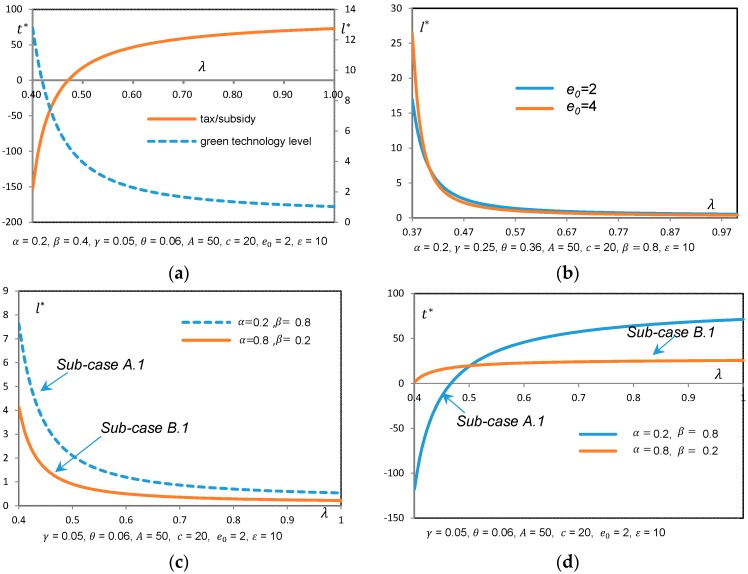
(**a**) $l^{*}$ and $t^{*}$ w.r.t. λ in sub-case A.1; (**b**) 𝑙^*^ w.r.t. *λ* for different values of *e*_0_ in sub-case A.1; (**c**) $l^{*}$ w.r.t. *λ* in sub-cases A.1 and B.1; (**d**) $t^{*}$ w.r.t. *λ* in sub-cases A.1 and B.1.

**Figure 2:**
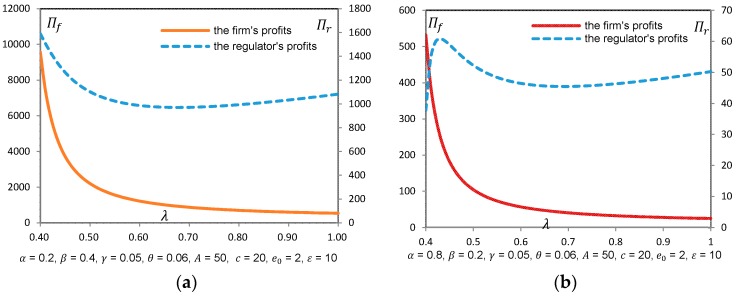
(**a**) $\Pi_{f}$ and $\Pi_{r}$ w.r.t. 𝜆 in sub-case A.1; (**b**) 𝛱_𝑓_ and 𝛱_𝑟_ w.r.t. 𝜆 in sub-case B.1.

Propositions 4 and 6 can be further explained with the help of Figure 1 and Figure 2.

Sub-case A.1 represents a market scenario, in which the ratio of the market’s elasticity of lower emissions to that of price is large enough to cover the extra cost (i.e., $\frac{\beta }{\alpha }\geq \frac{\theta }{\gamma }$ ), but the factors (such as β, γ, and *A*) motivating green technology investment are not so large (i.e., $\sqrt{\frac{\left(\beta \gamma -\alpha \theta \right)\left(A\gamma -\alpha \theta e_{0}\right)}{2\alpha ce_{0}}}<2$) that the firm needs to make a trade-off between the benefits and overall cost of green investment. In this case, the higher the regulator’s environmental concern, the lower the level of firm’s green investment (see Figure 1a). A reason behind this is that we assumed that the firm could observe the regulator’s environmental concern by what was being said and done, and could predict that a regulator with high level environmental concern adopts high tax rate, which, in turn, increases the overall cost of the firm with conditions given above. Thus, the firm will finally adopt low level green investment to response.

Sub-case B.1 demonstrate another market scenario, in which the ratio of the market’s elasticity of lower emissions to that of price is not large enough to cover the extra cost (i.e., $\frac{\beta }{\alpha }<\frac{\theta }{\gamma }$), but the firm can still find an optimal green technology level to maximize his profit if the difference between $\frac{\theta }{\gamma }$ and $\frac{\beta }{\alpha }$ is less than $2\varepsilon e_{0}$. In this case, similar to the discussion above, the firm’s green investment level decreases with increasing environmental concern of the regulator (see Figure 1a). However, the green investment level in Sub-case B.1 is lower than that in Sub-case A.1 (see Figure 1c)*.* This is intuitive since a more price-sensitive market offers less room for the firm to invest in green technology than a low-emission-sensitive market, and the firm cannot require a higher price for his effort of emission improvement in Sub-case B.1.

Conversely, a regulator with moderate environmental concern will adopt a relatively low tax rate, or even provide subsidy to the firm (see Figure 1a,d), which, in turn, leads to motivating green technology choice.

Sub-case A.2 represents a market scenario, in which the green-motivating factors (i.e., β, γ and *A*) are (individually or simultaneously) so large (i.e., $\sqrt{\frac{\left(\beta \gamma -\alpha \theta \right)\left(A\gamma -\alpha \theta e_{0}\right)}{2\alpha ce_{0}}}\geq 2$) that adopting highest level of green technology is always the best choice, regardless of the regulator’s environmental concern. However, Sub-case B.2 is exactly the opposite of Sub-case A.2, in which the green technology is too expensive to adopt (i.e., θ is large enough), or the firm faces an extremely price-sensitive market, and, therefore, the firm would have no encouragement to make any emission improvement in its production process.

Besides, we also found that $l^{*}$ increased with β, γ, ε, and *A*, and decreased with α, θ, and *c*, (the figures are not presented in the paper as the meanings are obviously clear ). However, Figure 1b shows somewhat difference implying the effect of $e_{0}$ on $l^{*}$. When comparing with low-$e_{0}$ case, a firm with higher $e_{0}$ will choose higher level green technology when facing a moderate λ, and will choose lower level green technology when facing an extremely higher λ (see Figure 1b). It is also shown from Figure 2a,b that social welfare may not necessarily increase when the regulator is extremely concerned with environmental impact. In this case, even though dirty technology is adopted, environmental benefits are obtained through lower consumption based on Proposition 3.

## 5. Conclusions

We investigated a three-stage game between a firm and a regulator, where the firm makes the long-run green technology investment in the first stage, the regulator sets its emission tax/subsidy rate in the second stage, and the firm choose its sale price in the final stage. We focused our research on the idea that what factors affect the level of green technology investment of the firm, and how these fators affect the investment. Thus, different cases with numerical example were investigated and discussed to generate insights that would inform policy making bodies and involved stakeholders. The following results are derived from our model and numerical analysis.

- High levels of the regulator’s environmental concerns do not necessarily lead to the choice of green technology. Our analysis demonstrated that, in a context that is not favorable to adopt green investment, high level of environmental concern results in overly high tax rates that, in turn, demotivate the firm from adopting the cleaner technology. However, if the regulator is moderately concerned with the environmental issue and therefore sets reasonable tax level or even subsidizes the firm, clean technology investment may be motivated and social welfare may be increased simultaneously.
- Green investment level depends on the combined effect of the market and operational factors for a given level of the regulator’s environmental concerns. Our results showed that when the consumers favor green products and are willing to pay extra costs for low-pollutant products, or the green technology is not so expensive to adopt, the firm may favor a high level of green investment. Otherwise, low levels of green technology are preferred. In two extreme cases when the market and operational factors satisfy certain conditions that we discussed above, the firm will always prefer highest level green investment or never invest in green technology at all.
- Increasing environmental awareness amongst the consumers is an effective way to drive the firm’s green investment. It was shown that whether the firm dedicates itself to green investment ultimately depends on how consumers think about environmental issues. In particular, the environmental awareness of consumers is relatively poor in developing countries, like China, where prices other than green features of products are the main factor influencing consumer purchases. As the result, the regulator in a developing country should strengthen publicity and education in various forms for better awareness of environmental issues amongst the consumers.

All of these results, however, should be interpreted in the scope of our models that focused on how the regulator’s environmental concern influences the firm’s green technology investment. Many implementation-related complexities of how to enable the firm to exactly understand the regulator’s environmental concerns were not taken into account by our stylized models. Moreover, administrative costs for designing optimal policy deserve closer scrutiny.

## Figures and Tables

**Figure A1:**
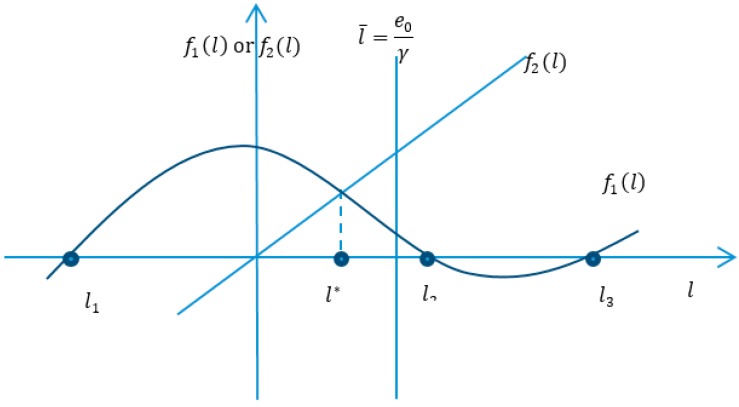
The curve of the function $f_{1}\left(l\right)\;$ or $f_{2}\left(l\right)$ in Sub-case A.1.

**Figure A2:**
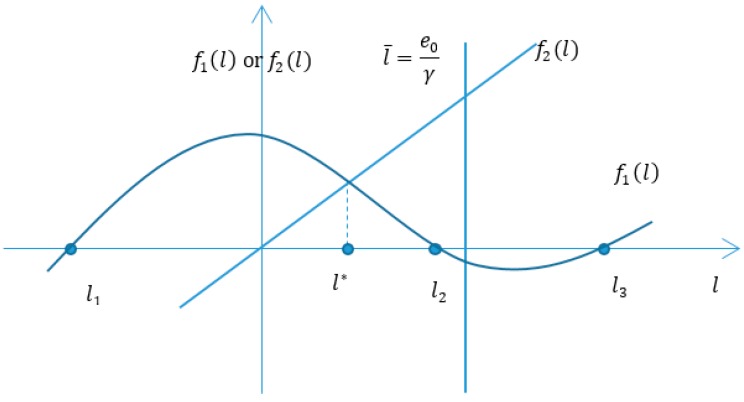
The curve of the function $f_{1}\left(l\right)\;\mathrm{or}\;f_{2}\left(l\right)$ in Sub-case B.1.

**Figure A3:**
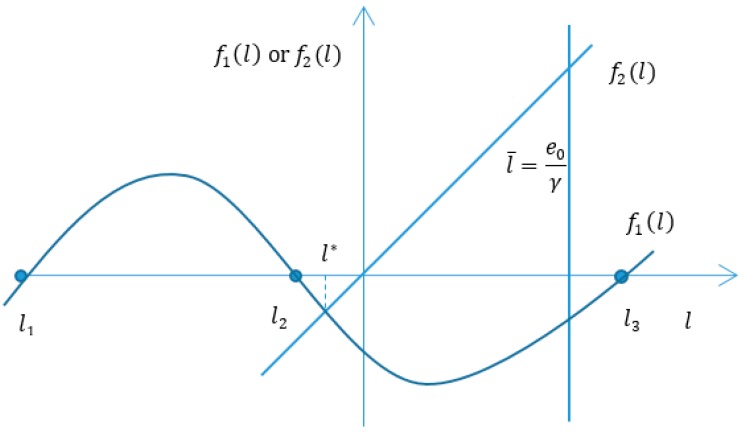
The curve of the function $f_{1}\left(l\right)\;\mathrm{or}\;f_{2}\left(l\right)$ in Sub-case B.2.

## Appendix A

**Proof of Proposition** **1.** The regulator maximizes $\Pi_{r}$ with respect to t, taking into account how t will affect the firm’s market response p. Substituting (5) into (3), the first-order condition is given by:

(A1) $$\frac{\partial \Pi_{r}}{\partial t}=\frac{S_{1}}{2}\left[\left(2\lambda -1\right)\left(A-\beta S_{1}-\alpha \theta l\right)+\lambda \alpha \varepsilon S_{1}^{2}-\left(3\lambda -1\right)\alpha S_{1}t\right]=0\;$$

where $S_{1}\equiv e_{0}-\gamma l$. (A1) yields unique second derivative as follows:

(A2) $$\frac{\partial^{2}\Pi_{r}}{\partial t^{2}}=-\frac{1}{2}\alpha \left(3\lambda -1\right)S_{1}^{2}$$

It is straightforward to see $\frac{\partial^{2}\Pi_{r}}{\partial t^{2}}>0$ when $0\leq \lambda <\frac{1}{3}$, implying that the regulator cannot find a unique $t^{*}$ to maximizes its profits without a budget limit if it exerts an extreme concern on economic development in this case.

## Appendix B

**Proof of Proposition** **4.** Taking the first derivative of the firm’s profit, $\Pi_{f}$ which is given by (11), with regard to the firm’s technology level, l, we have

(A3) $$\frac{\partial \Pi_{f}}{\partial l}=\frac{\lambda^{2}\left(S_{2}+S_{3}l-\alpha \varepsilon \gamma^{2}l^{2}\right)\left(S_{3}-2\alpha \varepsilon \gamma^{2}l\right)-2\alpha c{\left(3\lambda -1\right)}^{2}l}{2\alpha {\left(3\lambda -1\right)}^{2}}\;$$

(A4) $$\frac{\partial^{2}\Pi_{f}}{\partial l^{2}}=\frac{\lambda^{2}\left(6\alpha^{2}\varepsilon^{2}\gamma^{4}l^{2}-6\alpha \varepsilon \gamma^{2}S_{3}l+S_{3}^{2}-2\alpha \varepsilon \gamma^{2}S_{2}\right)-2\alpha c{\left(3\lambda -1\right)}^{2}}{2\alpha {\left(3\lambda -1\right)}^{2}}\;$$

Defining $f_{1}\left(l\right)=\lambda^{2}\left(S_{2}+S_{3}l-\alpha \varepsilon \gamma^{2}l^{2}\right)\left(S_{3}-2\alpha \varepsilon \gamma^{2}l\right)=0$, we can obtain three roots as

$$\{\begin{array}{c} l_{1}=\frac{S_{3}-\sqrt{S_{3}^{2}+4\alpha \varepsilon \gamma^{2}S_{2}}}{2\alpha \varepsilon \gamma^{2}}\\ l_{2}=\frac{S_{3}}{2\alpha \varepsilon \gamma^{2}}\\ l_{3}=\frac{S_{3}+\sqrt{S_{3}^{2}+4\alpha \varepsilon \gamma^{2}S_{2}}}{2\alpha \varepsilon \gamma^{2}} \end{array}$$

where $l_{1}<0$ and $l_{3}>l_{2}>0$. It is easy to prove that $\frac{\partial^{2}\Pi_{f}}{\partial l^{2}}<0$ when $0<l<l_{2}$ with previous assumptions in Section 3 and Section 4. Defining $f_{2}\left(l\right)=2\alpha c{\left(3\lambda -1\right)}^{2}l$, we assumed there existed a $l^{*}$ which satisfied $f_{1}\left(l^{*}\right)=f_{2}\left(l^{*}\right)$ and $0<l^{*}<l_{2}$ (as shown in Figure A1). Then the first and second order conditions were both satisfied (i.e., $\frac{\partial \Pi_{f}}{\partial l^{*}}=0,\frac{\partial^{2}\Pi_{f}}{\partial l^{*2}}\;<0$).

As show in Figure A1, we had $l_{2}-\bar{l}=\frac{S_{3}}{2\alpha \varepsilon \gamma^{2}}-\frac{e_{0}}{\gamma }=\frac{\beta \gamma -\alpha \theta }{2\alpha \varepsilon \gamma^{2}}\geq 0$. Thus, $l^{*}\in \left[0,\bar{l}\right]$ is satisfied only if

(A5) $$f_{2}\left(\bar{l}\right)-\;f_{1}\left(\bar{l}\right)=\frac{2\alpha ce_{0}{\left(3\lambda -1\right)}^{2}-\lambda^{2}\left(\beta \gamma -\alpha \theta \right)\left(A\gamma -\alpha \theta e_{0}\right)}{\gamma }>0\;$$

It is straightforward that (A5) holds only if $\frac{1}{3-\sqrt{\frac{\left(\beta \gamma -\alpha \theta \right)\left(A\gamma -\alpha \theta e_{0}\right)}{2\alpha ce_{0}}}}<\lambda <1$ and $\sqrt{\frac{\left(\beta \gamma -\alpha \theta \right)\left(A\gamma -\alpha \theta e_{0}\right)}{2\alpha ce_{0}}}<2$.

## Appendix C

**Proof of Proposition** **5.** Based on Appendix B, $f_{2}\left(\bar{l}\right)-\;f_{1}\left(\bar{l}\right)\leq 0\;$ if $\sqrt{\frac{\left(\beta \gamma -\alpha \theta \right)\left(A\gamma -\alpha \theta e_{0}\right)}{2\alpha ce_{0}}}\geq 2$. In this case, $\bar{l}\leq l^{*}<l_{2}$, meaning $l^{*}$ is the first-best choice but not a feasible solution if $l^{*}>\bar{l}$. Thus the best choice in this case is $\bar{l}$.

## Appendix D

**Proof of Proposition** **6.** In this case, it is straightforward to see that $S_{3}\equiv \beta \gamma -\alpha \theta +2\alpha \varepsilon e_{0}\gamma >0$ and $0<l_{2}<\bar{l}<l_{3}$ based on Appendix B. It is shown in Figure A2 that there existed a $l^{*}$ which satisfied $f_{1}\left(l^{*}\right)=f_{2}\left(l^{*}\right)$ and $0<l^{*}<l_{2}$. Therefore, the first and second order conditions were both satisfied (i.e., $\frac{\partial \Pi_{f}}{\partial l^{*}}=0,\frac{\partial^{2}\Pi_{f}}{\partial l^{*2}}<0$) for any $\;\lambda \in \left[\frac{1}{3},1\right]$ in this case.

## Appendix E

**Proof of Proposition** **7.** In this case, we had $S_{3}\equiv \beta \gamma -\alpha \theta +2\lambda \alpha \varepsilon e_{0}\gamma <0$, $l_{2}=\frac{S_{3}}{2\lambda \alpha \varepsilon \gamma^{2}}<0<\bar{l}$, and $l_{3}>\bar{l}$ based on (10). Thus, the optimal solution of l which satisfied $f_{1}\left(l^{*}\right)=f_{2}\left(l^{*}\right)$ (i.e., $\frac{\partial \Pi_{f}}{\partial l^{*}}=0,\;\frac{\partial^{2}\Pi_{f}}{\partial l^{*2}}<0$), however, fell outside the feasible region of l (as shown in Figure A3). Thus, the best feasible choice in this case was $l^{*}=0$.

## References

[1] Fan Q., Zhou X., Zhang T. (2016). Externalities of dynamic environmental taxation, paths of accumulative pollution and long-term economic growth. Econ. Res. J..

[2] Isik M. (2004). Incentives for Technology Adoption under environmental policy uncertainty: Implications for green payment programs. Environ. Resour. Econ..

[3] Dewit G., Leahy D. (2015). Tax uniformity: A commitment device for restraining opportunistic behavior. J. Public Econ. Theory.

[4] Abbey J.D., Meloy M.G., Guide V.D.R., Atalay S. (2015). Remanufactured products in closed-loop supply chains for consumer goods. Prod. Oper. Manag..

[5] Agrawal V., Atasu A., Van Ittersum K. (2015). Remanufacturing, third-party competition, and consumers’ perceived value of new products. Manag. Sci..

[6] Esenduran G., Kemahliogluziya E. (2015). A comparison of product take-back compliance schemes. Prod. Oper. Manag..

[7] Ovchinnikov A. (2011). Revenue and cost management for remanufactured products. Prod. Oper. Manag..

[8] Ovchinnikov A., Blass V., Raz G. (2014). Economic and environmental assessment of remanufacturing strategies for Product + Service Firms. Prod. Oper. Manag..

[9] Raz G., Druehl C.T., Blass V. (2013). Design for the environment: Life-cycle approach using a newsvendor model. Prod. Oper. Manag..

[10] Subramanian R., Ferguson M.E., Toktay L.B. (2013). Remanufacturing and the component commonality decision. Prod. Oper. Manag..

[11] Du S., Hu L., Wang L. (2015). Low-carbon supply policies and supply chain performance with carbon concerned demand. Ann. Oper. Res..

[12] Rezaee A., Dehghanian F., Fahimnia B., Beamon B. (2017). Green supply chain network design with stochastic demand and carbon price. Ann. Oper. Res..

[13] Sarkis J. (2015). Policy insights from a green supply chain optimisation model. Int. J. Prod. Res..

[14] Tseng S.C., Hung S.W. (2014). A strategic decision-making model considering the social costs of carbon dioxide emissions for sustainable supply chain management. J. Environ. Manag..

[15] Wang K., Zhao Y., Cheng Y., Choi T.M. (2014). Cooperation or competition? Channel choice for a remanufacturing fashion supply chain with government subsidy. Sustainability.

[16] Gsottbauer E., Den Bergh J.C.J.M. (2014). Environmental policy when pollutive consumption is sensitive to advertising: Norms versus status. Ecol. Econ..

[17] Lee S., Park S., Kim T. (2015). Review on investment direction of green technology R&D in Korea. Renew. Sustain. Energy Rev..

[18] Lambertini L., Tampieri A. (2015). Overcompliance with endogenous environmental standards and quantity competition. J. Environ. Econ. Policy.

[19] Espínola-Arredondo A., Muñoz-García F. (2016). Profit-enhancing environmental policy: Uninformed regulation in an entry-deterrence model. J. Regul. Econ..

[20] Bian J., Guo X., Li K.W. (2017). Decentralization or integration: Distribution channel selection under environmental taxation. Transp. Res. Part E.

[21] Krass D., Nedorezov T., Ovchinnikov A. (2013). Environmental taxes and the choice of green technology. Prod. Oper. Manag..

[22] Arguedas C., Cabo F., Martinherran G. (2016). Optimal pollution standards and non-compliance in a dynamic framework. Environ. Resour. Econ..

[23] Bi G., Jin M., Ling L., Yang F. (2016). Environmental subsidy and the choice of green technology in the presence of green consumers. Ann. Oper. Res..

[24] Barnett A.H. (1980). The Pigouvian tax rule under monopoly. Am. Econ. Rev..

[25] Requate T. (2005). Environmental Policy under Imperfect Competition: A Survey.

[26] Atasu A., Ozdemir O., Van Wassenhove L.N. (2013). Stakeholder perspectives on e-waste take-back legislation. Prod. Oper. Manag..

[27] Dong C., Shen B., Chow P., Yang L., Ng C.T. (2016). Sustainability investment under cap-and-trade regulation. Ann. Oper. Res..

[28] Laroche M., Bergeron J., Barbaro Forleo G. (2001). Targeting consumers who are willing to pay more for environmentally friendly products. J. Consum. Mark..

[29] Roheim C.A., Asche F., Santos J.I. (2011). The elusive price premium for ecolabelled products: Evidence from seafood in the UK market. J. Agric. Econ..

[30] Jones R., Mendelson H. (2011). Information goods vs. industrial goods: Cost structure and competition. Manag. Sci..

